# Disinhibited Attachment Disorder in UK Adopted Children During Middle Childhood: Prevalence, Validity and Possible Developmental Origin

**DOI:** 10.1007/s10802-016-0131-2

**Published:** 2016-02-09

**Authors:** Catherine Kay, Jonathan Green, Kishan Sharma

**Affiliations:** 1 Institute of Brain Behaviour and Mental Health, University of Manchester, Manchester, UK; 2 Room 3.316, Jean McFarlane Building, University of Manchester, Oxford Road, Manchester, England M13 9PL UK; 3 Pennine Care NHS Foundation Trust, Manchester, UK

**Keywords:** Attachment disorder, Maltreatment, Adoption, Externalizing psychopathology

## Abstract

We investigate the prevalence, specificity and possible aetiology of Disinhibited Attachment Disorder (DAD) in adopted children without a history of institutional care. Sixty children adopted from UK out-of-home care (AD; mean age 102 months, 45 % male); 26 clinic-referred children with externalizing disorder (ED; mean age 104 months, 77 % male) but no history of maltreatment or disrupted care; and 55 matched low-risk comparison controls (LR; mean age 108 months, 49 % male) were assessed for DAD using a triangulation of parent, teacher, and research observations. Maltreatment history and child psychiatric symptoms were obtained from parent report and child language development was assessed. DAD was identified in 49 % of AD, 4 % of ED and 6 % of LR children. Seventy-two percent of AD children had suffered maltreatment. DAD was not associated with degree of risk exposure, demographics, or language. A significant association with ADHD did not explain variance in DAD prevalence across groups. DAD was significantly more common in children first admitted to out-of-home care between 7 and 24 months, independent of maltreatment severity, age at adoption and number of care placements. Implications for developmental theory, adoption policy and clinical application are discussed.

**Electronic supplementary material:**

The online version of this article (doi:10.1007/s10802-016-0131-2) contains supplementary material, which is available to authorized users.

The concept of attachment disorder is one of the least evidenced psychiatric nosologies. Original criteria were influenced by a small longitudinal study following the adjustment of children in UK orphanages (Tizard and Rees 1975). Two patterns of behavior were identified; one of indiscriminate sociability with familiar and unfamiliar adults, disinhibition, attention seeking and excessive clinginess; and another of emotional withdrawal and unresponsiveness. Both were thought to arise as a direct result of social deprivation from caregivers who were discouraged from forming individual-child relationships. The two behaviour patterns were subsequently codified into Disinhibited Attachment Disorder (DAD) and Inhibited Attachment Disorder (IAD) and introduced to DSM-III (American Psychiatric Association 1980) under the overall descriptor of Reactive Attachment Disorder (RAD).

Very little further research followed until the late 1980’s when a number of children from severely depriving Romanian institutions were adopted to families in the UK, USA and Canada. A set of longitudinal studies ensued generating the first large-scale systematic evidence of Attachment Disorder in children adopted after severe early social deprivation (Bruce et al. 2009; Chisholm et al. 1995; O’Connor et al. 2000). The UK study followed a cohort of 165 adoptees and indexed three parent-report items indicative of DAD: the propensity to go off with a stranger, a lack of checking back with parents and a lack of differentiation between attachment figures. At 6 years old, 22 of 141 (15.6 %) children showed significant problems across all three of these items and 67 of 141 (47.5 %) had isolated symptoms, with substantive stability from age 4 (O’Connor et al. 2000). At 11 years, persistent DAD was found in 39.1 % of the sample (Kumsta et al. 2010); with further continuity through to 15 years in 29/42 (69 %) of these cases, usually in combination with some degree of quasi-autistic social impairment, major functional impairment and in service use (Kreppner et al. 2010). Similar high rates of persistent indiscriminate friendliness were reported in post-institutionalized Romanian children adopted by Canadian families (Chisholm et al. 1995; Chisholm 1998).

DAD is now seen as a developmental disorder and was recast for DSM-5 as Disinhibited Social Engagement Disorder (DSED). Since this study predates the introduction of DSM-5 we retain the International Classification of Diseases (ICD; World Health Organization 1992) terminology for this paper although our data and conclusions also are applicable to the DSED definition. Several studies have demonstrated that DAD is not co-incident with attachment status; children with DAD can display a range of typical attachment patterns with their main caregivers in a stable environment (Chisholm 1998; Minnis et al. 2009; O’Connor et al. 2003). However, it is still assumed that the aetiology lies in limited opportunity for forming early selective attachment relationships. The precise mechanism of how DAD arises remains unclear and there is limited research on DAD occurring in environments beyond the institution. Important questions therefore remain concerning aetiology of DAD and whether the concept is generalizable to children’s development following familial maltreatment or neglect.

Moderate to high levels of indiscriminate friendliness (thought to represent a dimension of DAD) were reported in 42 of 93 (46 %) pre-school maltreated foster children compared to 11 of 60 (19 %) community comparisons (Pears et al. 2010). Indiscriminate friendliness was negatively associated with measures of inhibitory control (e.g., inhibition of attention and behavioral responses). Lyons-Ruth et al. (2009) found socially indiscriminate behavior to be moderately stable from 12 to 18 months (*r* (44) = .31), linked with maternal psychiatric history and teacher reported aggressive and hyperactive behavior at age 5. Kay and Green (2013) identified high levels (> 2 SD above low-risk means) of carer-reported DAD in 153 adolescents (mean age 174 months) in out-of-home substitute care placements after severe early maltreatment; including indiscriminate behavior (58 %; 89/153), superficiality in relationships (56 %; 86/153) and attention seeking (56 %; 86/153). Indiscriminate behavior - representing core features of DAD (asking strangers personal questions, propensity to wander away from caregivers and seeking comfort from strangers) - was associated with experience of multiple forms of maltreatment, earlier age-at-entry-to-care, psychopathology and increased impairment across multiple domains of functioning on independent researcher ratings of triangulated data sources. In further investigation of potential mechanisms of DAD within a sub-sample of maltreated adolescents (*n* = 63), Kay and Green (2015) found no association of indiscriminate behavior with performance on measures of social cognition, including theory of mind (ToM) and social information processing. Despite evidence of poor ToM and hostile biases in social information processing in the maltreated adolescents, it was concluded that socially indiscriminate behavior is unlikely to represent difficulty in interpretation of others’ actions or lack of recognition of social boundaries due to underlying social cognitive impairment and that alternative mechanisms should be considered.

There is very little research in addition to that noted above on DAD in maltreated samples beyond the pre-school years and comparison of findings across existing studies is limited by the use of variable definitions and measurement. Most previous studies of non-institutionalized samples have used only parent report of DAD behavior and none focus exclusively on the middle childhood years - when children are most likely to first present to mental health services. Studies using multi-informant cross-context measures that incorporate observational methods are needed to fully assess the prevalence of DAD in maltreated samples across development. In this context, the first aim of this study was to assess DAD during middle childhood in maltreated non-institutionalized children using rigorous multi-informant methodology.

Another unresolved question concerns the specificity of DAD in non-institutional contexts. Since familial maltreatment is highly associated with multiple forms of co-occurring psychopathology a parsimonious hypothesis could be that DAD might represent a combination of symptoms associated with high-risk exposure, including disinhibition and relationship difficulties, which are features for example of Attention-Deficit/Hyperactivity Disorder (ADHD) and Conduct Disorder (CD). A second aim of this study is to test whether DAD is indeed an identifiable specific syndrome not simply explained by a combination of co-morbid forms of externalizing psychopathology in high-risk children. If DAD is an outcome specific to the experience of early adversity and/or disrupted care we may expect to see limited evidence of DAD in children without such experience, regardless of the presence of other forms of psychopathology. We test this using an age-matched comparison cohort with identified externalizing psychopathology of this type, where co-morbidity is likely to be common, but with no experience of maltreatment or disrupted care.

To summarize, we aimed to assess DAD during middle childhood in children adopted from UK out-of-home care (foster and other forms of substitute care) using rigorous multi-informant methodology and to examine association with early adversity and concurrent psychopathology. We tested the hypotheses that; i) children adopted from UK out-of-home care will have experience of pre- and post-natal adversity, including maltreatment and disrupted care; ii) adopted children will show higher prevalence of DAD than children who have not experienced substitute care or maltreatment; iii) DAD will be specific to a maltreatment/disrupted care context and will not be explained as a combination of other psychopathology, indicated by lower prevalence in a comparison group with identified externalizing disorders but no experience of substitute care or maltreatment; iv) presence of DAD will be associated with other forms of psychopathology and; v) more common in those with higher severity of adverse and disrupted care experience.

## Method

### Samples

#### Adoptive Sample (AD)

Sixty children aged 6 to 11 years, adopted from UK out-of-home care were recruited through Adoption UK, a national membership charity for adoptive families. The study was advertised on the Adoption UK website and in their literature as a study of social outcomes after adoption, with neither DAD nor any specific hypotheses mentioned. Families volunteered to take part by contacting the research team. Sample characteristics are detailed in Table 1.

**Table 1:** Sample characteristics: demographics, education and psychopathology

Variable	Sample			Test Statistic	
	AD (*N* = 60)	ED (*N* = 26)	LR (*N* = 55)		
Age *M* (*SD*)					
				*F*	*df*
	102 (20)	104 (29)	108 (21)	.956	2
Gender *n* (%)					
				χ^2^	*df*
Male	27 (45)	20 (77)	27 (49)	7.83*	2
Female	33 (55)	6 (23)	28 (51)		
Ethnicity					
White british	50 (83)	24 (92)	51 (93)	10.61	6
Mixed white and black	7 (12)	0	1 (2)		
Mixed white and asian	1 (2)	2 (8)	1 (2)		
Other mixed	2 (3)	0	2 (3)		
Parent educational level					
None	0	0	1 (2)	61.88***	6
Secondary	2 (3)	15 (58)	3 (6)		
Post-16	2 (3)	3 (11)	3 (6)		
Higher Degree/professional	55 (93)	5 (19)	43 (86)		
Educational placement					
Mainstream	58 (97)	26 (100)	55 (100)		
SEN	2 (3)	0	0		
Learning disability (%)	7 (12)	0	0		
Language M (*SD*)					
				*F*	*df*
Word classes	26.6 (9.6)	22.5 (10.4)	27.9 (8.9)	2.78	2
Recalling sentences	50.8 (16.8)	49.9 (12.6)	64.6 (14.5)	13.8***	2
Psychopathology *n* (%)					
(Total N)	(54)	(13)	(37)		
Any disorder	35 (65)	13 (100)	7 (19)		
Emotional disorder	17 (32)	4 (31)	3 (6)		
ADHD	24 (44)	10 (77)	1 (2)		
ODD	21 (39)	5 (38)	2 (4)		
CD	6 (11)	5 (38)	1 (2)		

AD = adopted; ED = externalizing disorder; LR = low risk. SD = standard deviation; df = degrees of freedom; SEN = special educational needs; *M* = mean. ADHD = attention deficit hyperactivity disorder; ODD = oppositional defiance disorder; CD = conduct disorder

*p < .05

***p < .001

#### Externalizing Disorder (ED) Sample

Twenty-six children were recruited from three Child and Adolescent Mental Health Services (CAMHS) in Greater Manchester. Children aged 6–11 years and living with one or both birth parents were screened by clinicians for current externalizing behavioral difficulties indexed by a raw score of three or more out of 10 on five Strengths and Difficulties Questionnaire (SDQ) conduct problem items (Goodman et al. 2000a), and/or five or more out of 16 on eight additional items indexing DSM criteria for Oppositional Defiance Disorder (ODD; American Psychiatric Association 2013). The scores represent established cut-offs used to identify children from a large UK school based population (*n* = 3675) scoring within the top 15 % on parent and teacher reported conduct problems (Scott et al. 2012). The scales have been shown to significantly predict variance on independent measures of anti-social behavior (Scott et al. Personal Communication). To control for early adverse experience and disrupted care, exclusion criteria included a history of being looked after by the local authority, adopted or social services involvement with the family and/or diagnosed developmental disorder. Potential participants were screened by CAMHS mental health clinicians using SDQ conduct problem and ODD scores and information regarding diagnoses, family composition, care history and risk. History of local authority care, adoption and/or social services involvement with the family was also confirmed via parent report. Thirty-five eligible children were referred and 26 consented to take part. None of these included children were found to have experience of local authority care, adoption or social services involvement following referral.

#### Low-Risk (LR) Sample

Fifty-five community comparison children with no history of being looked after by the local authority, adopted or social services involvement for child protection concerns were recruited from mainstream primary schools in Greater Manchester, and matched to the AD sample for age, gender, ethnicity and adoptive family socio-economic status (SES). Children attending CAMHS at the time of recruitment were excluded (*n* = 1).

Exclusion criteria for all samples were if a parent (including adoptive) reported i) moderate to severe learning disability, ii) poor spoken English or iii) current severe mental health problem (e.g., psychosis).

### Measures

#### Maltreatment and Care History in the AD Sample

Data on maltreatment and care history was collected from adoptive parent report, including the child’s age-at-entry to care, number of placements, age at adoption, detail of known physical, sexual, emotional abuse and/or neglect and pre-care experience. Severity ratings of parents’ verbatim descriptions were made based on the coding anchors of the Maltreatment Classification System (MCS; Barnett et al. 1993) noting the severity or harshness of the reported act of the caregiver (e.g., leaving a 8 year old child to care for pre-school age siblings), and physical outcome for the child (e.g., minor burns or treatment for malnutrition) is rated. A severity rating of zero to five was made for descriptions of emotional maltreatment, lack of supervision, failure to provide for the child, physical maltreatment and sexual abuse. One rating was given for each category reflecting the most severe episode of abuse. A score of zero was given when there was no report of incidents or evidence relating to a maltreatment category. To control for variability in the knowledge of parents, a confidence rating of zero to two was assigned to each rating; 0 = *parent acknowledged a lack of information*; 1 = *poor detail in descriptions*; 2 = *detailed descriptions of specific events and evidence*. The ratings for each form of maltreatment were summed to produce a single maltreatment severity score ranging from 0 to 25. A second researcher rated all cases, blind to the scores of the first rater and agreement was excellent (*ICC* = 0.89).

Evidence of pre-natal exposure to adversity (e.g., drug or alcohol misuse during pregnancy or complications during birth) was rated as present or absent from adoptive parents’ report of pre-care experiences. In cases where pre-natal exposure was suspected but not confirmed pre-natal adversity was coded as present.

#### Disinhibited Attachment Disorder (DAD)

DAD was assessed using triangulated multi-informant data from parents, teachers and researcher observation. There are no gold-standard assessments for DAD. We therefore selected validated measures that index core features of DAD consistent with ICD-10 criteria and previously reported in maltreated and institutionalized samples (Zeanah and Gleason 2010). The Child and Adolescent Psychiatric Assessment-Reactive Attachment Disorder (CAPA-RAD; Minnis et al. 2009) was administered with parents. The CAPA-RAD is a semi-structured interview consisting of 22 items assessing ICD-10 diagnostic criteria for DAD, which has been found to discriminate 98 % of attachment disorder cases from low-risk comparisons and which has good test-retest reliability (Minnis et al. 2009). Six items identify DAD in CAPA-RAD and are consistent with DSM-5 specifications for DSED; indiscriminate relationships with adults, cuddliness with strangers, asking personal questions of adults, attention seeking behavior (consistent with ICD-10 criteria for DAD in older children), lack of checking back with the caregiver and lack of awareness of social boundaries. The data from these items are used in this study. Each behavior is rated as present or absent based on parents’ responses to a series of standard questions and prompts.

The Relationship Problems Questionnaire (RPQ; Minnis et al. 2009) was completed by a teacher of participating children. The RPQ was developed alongside the CAPA-RAD as part of a multi-informant assessment of RAD. The RPQ has been used in several large-scale studies of attachment disorder in children and adolescents (Minnis et al. 2007; Pritchett et al. 2013). Teachers are asked to rate 14 statements on a four point scale (0 = *not at all like child*, 1 = *a bit like*, 2 = *like*, 3 = *exactly like child*). DAD items are; often asks very personal questions, gets too physically close to strangers, is too cuddly with people s/he does not know, and is too friendly with strangers (α = .92).

Researcher observation of child disinhibition was completed following a 2.5-hour home visit with the child on a standard instrument that was previously used to identify DAD in post-institutionalized samples (Rutter et al. 2007). Standardized ratings of the presence of the following were made; general disinhibition, (e.g., treats the examiner as if they were a close friend); unsolicited physical contact and close physical proximity (e.g., child sits in experimenter’s lap or hugs experimenter); violation of verbal boundaries (e.g., constant talking or intrusive questioning); and violation of social boundaries (e.g., removing clothing in front of the researcher). The researcher noted the child’s behavior towards them throughout the home-visit and indicated the presence of each form of behavior as *present* (1) or *absent* (0).

#### Psychopathology

The online Development and Wellbeing Assessment (DAWBA; Goodman et al. 2000b) was used to assess psychopathology. The DAWBA is an extensively validated web based parent-report questionnaire consisting a series of fixed choice response (does your child worry?) and open ended (what does your child worry about?) questions regarding behavior and symptoms associated with DSM-IV diagnostic criteria for a range of emotional, behavioral, developmental and hyperactivity disorders. A computer program uses captured information to predict likelihood, within six probability bands ranging from <0.1 % to >70 % chance, of meeting DSM-IV criteria for each diagnostic category. A trained clinical rater reviews all evidence, including open-ended responses to accept or override the computer-generated diagnoses. The clinical reviewer’s decision ratings (*present* = 1 or *absent* = 0) for each diagnostic category were used in analysis. The DAWBA has been used in a number of large-scale epidemiological studies to assess mental health needs of children in the UK (Meltzer et al. 2000; Ford et al. 2007) and the diagnostic algorithms have been developed based on analysis and independent clinical review of over 20,000 cases in international survey studies.

#### Language Ability

Children completed the Word Classes and Sentence Recall subtests of the Clinical Evaluation of Language Fundamentals-4 (CELF-4; Semel et al. 2003). These subtests have been widely used and correlate highly with expressive (*r* = .81) and receptive (*r* = .85) language scores derived from multiple other subtests of the CELF-4 (Botting and Conti-Ramsden 2008).

### Procedure

Parents and children were assessed during a home-visit. Parents completed questionnaires on demographic information, the child’s experience of maltreatment and care history; they then undertook a researcher interview for the CAPA-RAD. The researcher undertook the in-person child assessments including the CELF; and made structured observational ratings using the DAD observation schedule throughout the visit, which typically lasted for 2.5 hours. Parents completed the DAWBA online after the visit, with back-up telephone support as necessary and periodic prompts for completion. The teacher questionnaire was sent in the post for completion and return.

### Analysis

Descriptive statistics were used to explore experience of maltreatment and care history in the AD sample including type and severity of maltreatment, age-at-entry to care and rates of pre-natal exposure to adversity. To assess prevalence and specificity of DAD across samples in order to test the hypothesis that DAD will be found at higher rates in the adopted sample and specific to the maltreatment/disrupted care context, we use triangulation of CAPA-RAD, RPQ and Observation measures. Due to a floor effect in RPQ data and to aid in triangulation of data, the RPQ scores were collapsed to produce a binary score for each item (*not at all like child* =0, *a bit like, like or exactly like child* =1). Participants with two or more reported symptoms (corresponding to individual items) on two or more different informant measures were rated as *DAD present* (1), whilst those scoring less than two on two measures or two or more on only one measure were rated as *DAD absent* (0). Validity of the resultant binary DAD variable was assessed through association with continuous measures of DAD. The DAD variable was subsequently used to test the remaining hypotheses that presence of DAD will be associated with increased rates of psychopathology and severity of maltreatment and adverse experience. A series of binary logistic regression analyses were performed with DAD as a predictor of DAWBA disorders, adjusting for age, gender and ethnicity. Association of DAD with maltreatment and care history variables was tested in a series of binary logistic regression analyses with DAD entered as the dependent variable, adjusting for age, gender and ethnicity.

## Results

### Sample Characteristics

#### Matching

Sample characteristics are shown in Table 1. The three samples were well matched for age and ethnicity. AD and LR samples were well matched on gender, χ^2^(1) = .193 and parental educational level, χ^2^(3) = 2.141. However, across the three groups a higher proportion of the ED group were male and a lower proportion of ED group parents had a higher degree or professional qualification than the adoption and LR sample (Table 1). All ED and LR participants were in mainstream education and none had parent reported learning disability. Two AD children were in a special educational needs (SEN) placement and seven were reported to have a learning disability.

#### Language

There was no significant difference in CELF Word Classes scores between the adopted and LR or ED samples before or after controlling for age, gender and ethnicity in multiple regression analysis, using dummy variables to represent sample groups, but the LR group scored significantly higher than the ED group, *B* = −5.24, *SE* = 2.22, *p* = .020. The LR group also scored significantly higher on the Recalling Sentences CELF subtest than the ED and AD samples, *B* = −12.96, *SE* = 3.12, *p* = .000 and *B* = −12.801, *SE* = 2.44, *p* = .000, respectively (Table 1).

#### Psychopathology

Sixty five percent (35/54) of the AD sample and 19 % (7/37) of the LR sample met threshold criteria for any psychiatric disorder on the DAWBA (Table 1). DAWBAs were returned for only half (*n* = 13) of the ED sample. There were no significant differences in the age, *t*(24) = −1.217, gender, χ^2^(1) = .000, ethnicity, χ^2^(1) = .000, or parent educational level, χ^2^(2) = .558 of participants in the ED sample with and without a DAWBA. The entire ED sample for which DAWBA was available met criteria for a psychiatric disorder. Similar rates of psychopathology were present in the AD and ED samples, with no significant difference in presence of ADHD, ODD or CD. Twenty percent (11/54) of the AD sample showed co-morbidity of ADHD, emotional and CD or ODD. A further 20 % showed co-morbidity of two disorders: commonly CD or ODD and ADHD, compared to 61.5 % (8/13) of the ED sample showing co-morbid CD or ODD and ADHD, and 15 % (2/13) showing co-morbid emotional disorder and ADHD. By contrast the LR sample showed significantly lower rates of ADHD, *OR* = .008, *p* ≤ .000, ODD, *OR* = .098, *p* < .05 and CD/ODD, *OR* = .056, *p* < .05, controlling for age, gender and ethnicity. There were no significant differences in rates of emotional disorder between the three groups.

### Maltreatment and Care History in the AD Sample

Seventy two percent (43/60) of AD children had been exposed to maltreatment and 53 % (32/60) had experienced more than two forms of maltreatment. Table 2 shows details of category and severity of maltreatment experience. Fifty-five percent of AD children experienced pre-natal adversity (e.g., exposure to pre-natal maternal drug or alcohol misuse) (18/60 [30 %] suspected exposure, 6/60 [10 %] documented exposure, 9/60 [15 %] with physical symptoms at birth which required treatment). Six (10 %) had no known experience of either pre or post-natal exposure to adversity; 22/60 (37 %) had both pre and post-natal exposure; 21/60 (35 %) experienced just post-natal and 11/60 (18 %) had just pre-natal exposure. Mean age at entry to out-of-home care was 12.5 months, *SD* = 15.6; range 0 to 60 months. All children entered care due child protection concerns about actual or potential harm. Twenty children (33 %) entered care at birth (Table 2). Mean number of care placements was 3.1, *SD* = 1.5; range 2–10, and mean length of time spent in out-of-home care prior to adoption was 24.3 months, *SD* = 22.2. Mean age at adoption was 35.5 months, *SD* = 27. All children were adopted from foster care and one child was adopted in the first month but all experienced an out-of-home care placement. Three children were adopted before the age of 6 months and a further seven by 12 months. There was a significant negative correlation between maltreatment severity scores and confidence ratings, −.406, *p* < .01, where lower confidence ratings were associated with higher severity scores, reflecting the high rates of confidence in maltreatment information on children who were admitted to out-of-home care at birth. When age at first admission to out-of-home care is controlled, the correlation falls to −.162 and is no longer significant.

**Table 2:** Maltreatment variables and age at first entry to out-of-home care in the adopted sample

Maltreatment Category	Statistics			
			Severity rating of maltreatment Category	
	N	(%)	*M*	(*SD*)
Emotional	40	(68)	2.1	(1.8)
Physical	18	(30)	0.4	(0.8)
Sexual	9	(15)	0.1	(0.4)
Neglect (FtP)	38	(64)	1.1	(1.3)
Neglect (LoS)	36	(60)	1.7	(1.8)
Total Severity			5.5	(4.6)
Grouped age at first admission to care (months)				
Age group	N	(%)		
0	20	(33)		
1–6	11	(18)		
7–24	17	(28)		
> 25	12	(20)		

AD = adopted sample. *M* = mean; *SD* = standard deviation. FtP = failure to provide; LoS = lack of supervision

### Disinhibited Attachment Disorder (DAD)

#### Classification Validity

Internal consistency of summed DAD item scales from CAPA-RAD, RPQ and researcher observation were excellent, α = .825, .968 and .973, respectively. There was a negative skew with a floor effect in the distribution of continuous measures of DAD across all samples and significant positive correlations between all DAD measures (Supplementary Table 1). Eighty-one participants (81/113; 62 %) scored less than two on the CAPA-RAD, 88/111 (79 %) scored more than two on the RPQ and 97/129 (75 %) scored less than two on the observation. Validity of the categorical classification of DAD based on triangulation of measures (≥ two reported symptoms on ≥ two DAD measures = *DAD present*) was assessed against individual parent, teacher and researcher reports: all informant scores were significantly increased in cases of DAD (Supplementary Table 2). In ROC curve analysis predicting DAD, the CAPA-RAD and observation measure showed excellent sensitivity and specificity (area under the curve = .899 and .882 respectively), whilst the RPQ had acceptable sensitivity and specificity (area under the curve = .769).

#### Prevalence of DAD Across Samples

Classified DAD was present in 49 % (29/59) of the AD sample, 4 % (1/25) of the ED sample and 6 % (3/53) of the LR sample. A series of binary logistic regression analyses found no significant association between DAD caseness and age, gender, ethnicity, parental educational level, CELF language scores or parent reported learning disability (Table 3). In adjusted binary logistic regression and using the LR sample as the reference group, odds of DAD were significantly increased in the AD group, *OR* = 14.12, *p* ≤ .000, whilst there was no significant difference between the LR and CD group, OR = .408, ns, controlling for age, gender and ethnicity. There was no significant difference in prevalence of DAD in members of the ED sample with and without psychopathology data, χ^2^(1) = .962.

**Table 3:** Statistics from binary logistic regression analyses showing association of DAD with demographic variables across all samples and with DAWBA psychopathology, care history and maltreatment within the adopted sample

	Adjusted^a^ *OR*	95 % CI	
		*LL*	*UL*
Age	.980	.951	1.010
Gender	.791	.287	2.590
Ethnicity	3.78	.784	18.25
Parent educational level	.422	.031	5.803
Learning disability	.831	.118	5.827
Psychopathology^b^			
Any Disorder	.330	.105	1.031
Emotional	.317	.090	1.120
ADHD	.279*	.082	.949
ODD/CD	.307	.091	1.032
Care History			
Age at first admission to care (continuous)	.999	.964	1.035
Age-at-adoption	.997	.975	1.020
Number of care placements	1.16	.825	1.636
Maltreatment Severity			
Emotional abuse	1.11	.811	1.513
Sexual abuse	4.15	.726	23.69
Physical abuse	1.49	.725	3.057
Neglect (FtP)	1.28	.828	1.972
Neglect (LoS)	1.08	.789	1.465
Total maltreatment severity	1.07	.945	1.204
Pre-natal exposure	1.40	.463	4.230

DAWBA = development and wellbeing assessment. OR = odds ratio; CI = confidence interval; LL = lower limit; UL = upper limit. ADHD = attention deficit hyperactivity disorder; ODD/CD = oppositional defiance disorder/conduct disorder. FtP = failure to provide; LoS = lack of supervision

^a^All demographic variables were entered in to the model simultaneously

^b^Analyses controlled for age, gender and ethnicity

*p ≤ .05

#### Association of DAD with Psychopathology

AD children with DAD were significantly more likely to also show ADHD as measured on the DAWBA but no other forms of psychopathology in adjusted binary logistic regression controlling for age, gender and ethnicity (Table 3). Within the AD sample, individual CAPA-RAD, RPQ and observation measures suggested a pattern of response bias in reporting. Parents were more likely to show common reports of a range of psychopathology; CAPA-RAD scores predicted presence of DAWBA emotional disorder, *OR* = 1.644, *p* < .01; ADHD, *OR* = 1.792, *p* < .01 and CD/ODD *OR* = 1.711, *p* < .01. Teacher RPQ scores significantly predicted CD, *OR* = 1.630, *p* < .01. Researcher observation scores significantly predicted ADHD, *OR* = 1.95, *p* < .01.

#### Association of DAD with Maltreatment and Care History in the AD Sample

Within the AD sample, using a series of binary logistic regression analyses adjusted for age, gender and ethnicity, presence of DAD showed no significant independent association with care history (continuous measures of age at first admission to care, age at adoption and number of placement moves) or maltreatment history (severity of any individual form of maltreatment, pre-natal exposure to adversity or total maltreatment severity, before or after controlling for confidence rating; Table 3). However, presence of DAD did show a specific and unexpected association with child’s age at first admission to out-of-home care. Using age criteria established in the ERA study (O’Connor et al. 1999) within a binary logistic regression controlling for age, gender and ethnicity, and taking care-at-birth as a reference category, children who were first admitted to care between 7 and 24 months old were 9.9 times more likely to meet DAD criteria than those admitted at birth, whereas there was no significant odds of DAD in those admitted at 1–6 or over 25 months (Table 4). Using the 7–24 month group as the reference category, the odds of DAD were significantly lower in the groups admitted at birth, *OR* = .101, *p* < .01; 1–6 months, *OR* = .063, *p* < .01; and over 25 months, *OR* = .073, *p* ≤ .01.

**Table 4:** Statistics from binary logistic regression analysis showing relationship between grouped age at first entry to out-of-home care and presence of DAD in the adopted sample

Grouped age at first admission to care (months)	Total N	N (%) with DAD	Adjusted^*a*^ *OR*	95 % CI	
				*LL*	*UL*
0^b^	20	7 (37 %)			
1–6	11	4 (36 %)	.980	.108	3.597
7–24	17	14 (82 %)	9.890**	1.843	53.077
> 25	12	4 (33 %)	.721	.131	3.964

DAD = disinhibited attachment disorder. OR = odds ration; CI = confidence interval; LL = lower limit; UL = upper limit

^a^Analysis controlled for age, gender and ethnicity

^b^Entry to out-of-home care at 0 months was used as the reference category in this analysis

**p ≤ .01

Age at first entry to out-of-home care showed significant association with maltreatment severity, age at adoption and number of placements. Children who were first admitted to care at 0 months had significantly lower maltreatment severity scores than all other age at admission groups. Children admitted between 1 and 6 months had significantly lower scores than those admitted at 7–24 and >25 months, whilst there was no difference between the severity scores of those admitted at 7–24 and >25 months (Table 5). In logistic regression analysis controlling for grouped age at admission, maltreatment severity still did not predict DAD. This remained true after controlling for maltreatment confidence rating. Children who were admitted to care over 25 months were adopted significantly later than all other age at admission groups, *SS*(3) = 11,539, mean square = 3846, *F* = 7.120, *p* = .000, but age at adoption did not predict DAD after controlling for grouped age at admission to care. Children who entered care between 1 and 6 and 7–24 months experienced significantly more placement moves than those who entered care at 0 months, Mean diff = −1.52, *SE* = 0.53, *p* = .031 and Mean diff = −1.52, *SE* = 0.46, *p* = .011, respectively. Grouped age at admission to care remained a significant predictor of DAD after controlling for number of placements; whilst number of placements did not predict DAD.

**Table 5:** Statistics from ANOVA showing difference in mean maltreatment severity scores across age at first admission to out-of-home care groups

Grouped age at first admission to care (months)	Mean maltreatment severity (SD)	Contrast age at first admission to care group	MD
0	0.8 (2.1)	1–6	−4.29**
		7–24	−7.90***
		> 25	−8.45***
1–6	5.1 (2.8)	7–24	−3.61*
		> 25	−4.16**
7–24	8.7 (3.4)	> 25	−.544
> 25	9.2 (3.6)		

SD = standard deviation; MD = mean difference

*p ≤ .05

**p ≤ .01

***p ≤ .000

## Discussion

The aim of this study was to explore a number of key questions surrounding the concept of DAD when applied to a high-risk non-institutionalized sample. We examine; presence of DAD using multi-informant measures in a sample of children adopted from UK out-of-home care; construct validity and specificity of DAD to the maltreatment/disrupted care context independent of other forms of psychopathology via comparison with matched community and clinic referred samples; and potential developmental origin through tests of the relationship of DAD with severity of adverse experience and disrupted care history.

Our AD sample of children adopted from UK out-of-home care had commonly experienced multiple forms of maltreatment, pre-natal exposure to adversity and significant disruption in early care history, including multiple care placements prior to adoption. However, most had spent several years in the adoptive home at the time of assessment. A high percentage (49 %) of the AD sample met our criteria for DAD case-ness on triangulation of multi-informant parent, teacher and researcher measures. This is in contrast to the very low percentage (6 %) of matched low-risk community comparison children who also met these criteria. The rate of DAD in our AD sample is slightly higher, but nevertheless in line with rates of DAD previously shown in both adolescent post-institutionalized (O’Connor et al. 1999; Rutter et al. 2007) and young maltreated samples (Pears et al. 2010). It is slightly lower than the prevalence of 56 % reported in UK looked after adolescents (Kay and Green 2013); however this latter figure was based solely on carer report. It is not possible to rule out that the sampling method (self-referral via advertisement through an adoption charity) may have introduced sampling bias that inflates the identified rates of DAD in relation to possible rates across all adopted children. However, the background and demographics of our cohort is comparable to those of over 37,000 children subject to an adoption order in England between 2002 and 2011 (Selwyn et al. 2014) in terms of average age at admission to care, number of placements prior to adoption and rates of maltreatment. Rates of DAD were not examined in this, or other survey samples. However the comparability of these severity-related metrics suggests that a significant sampling bias is unlikely. Definitive answer to this question awaits a large survey study including DAD ascertainment.

Identification of DAD using a triangulated multi-informant methodology appears to maximize the construct validity and specificity of the identified DAD syndrome. While the parent-report only approach shows high levels of identified co-morbidity with other DAWBA symptomatology (as it did in work on DAD with maltreated adolescents; Kay and Green 2013), the triangulated DAD in this sample is only associated with carer reported ADHD. Comparison of psychopathology using parent-report measures, as is commonly done, introduces the likelihood of a common-reporter bias, which can inflate apparent co-morbidity. The triangulation method by contrast increases specificity and predictive validity. Data from our clinically referred comparison sample - selected to test syndrome specificity in DAD by excluding cases with known exposure to early adversity or disrupted care of the kind commonly thought to be associated with DAD - supports this. This ED comparison group shows very low rates of DAD using the triangulation method, although similar rates of other co-morbid psychopathology on DAWBA, most commonly CD and ADHD.

We next consider data relating to the potential developmental origins of DAD. From its inception, the concept of DAD has been linked to the hypothesized effect of disrupted early social relationships (Tizard and Rees 1975) and this continues through in the current DSM-5 formulation (APA, 2013). The adoptive sample in this study were selected as having had disrupted early care, high-risk of prior maltreatment and exposure to pre-natal adversity (e.g., maternal drug or alcohol misuse during pregnancy). Rates of reported maltreatment (73 %) and pre-natal adversity exposure (55 %) are in line with documented experiences of wider UK care populations (Department for Education 2013). Although a number of our adoptive sample entered the care system at birth, they too experienced disrupted care with movement from foster to adoptive placements. This group were identified very early on as being at risk of harm and had frequently experienced pre-natal adversity. Given the reported patterns of risk it is striking that DAD was not associated with maltreatment type, severity or extent of exposure; nor was it associated with pre-natal exposure. A strong association was however found with age at first admission to out-of-home care: children placed between 7 and 24 months old were nine times more likely to show DAD. Eighty-two percent of those entering care within this time frame showed DAD, compared to around 35 % of those placed at birth, before 6 months and after 2 years old. It should be noted that this association was observed only for DAD and no other form of psychopathology. This intriguing (and to us unexpected) finding is not sensitive to altering age criteria for grouping – indeed, all nine children admitted to care between 7 and 12 months old showed DAD. Nor is it explained by any measured independent factor or simple dose-response relationship, such as the influence of variables associated with age at admission to care including increased placement moves, differing levels of severity of maltreatment or age at adoption. This novel finding of course would need careful replication in independent samples before any firm inferences were drawn. We also acknowledge potential confounding from the data on maltreatment being gathered via adoptive parent report (the implications of this are discussed in further detail below) as well as limitations from the moderate sample size. Nevertheless, this preliminary finding as it stands, considered in the context of developmental theory and previous literature on DAD, raises at the least some key hypotheses for future testing.

Firstly, research documenting the normative development of selective attachments suggests a hypothesis that the finding could reflect disruption of primary selective attachments at some point during their initial development, generally accepted not to have formed before about 7 months, and having achieved more stable form after 2 years. In this explanation, the indiscriminate behavior characteristic of DAD might reflect a persistent failure to inhibit care-seeking behavior with adults who are not the primary caregiver; a failure in other words of the normal process of selective attachment due to the children being removed from their primary caregiver or experiencing disruption in care between 7 and 24 months of age. This formulation is consistent with a number of predictions. First, a persistent disinhibition of this kind in relation to strangers could co-exist with later-forming selective attachments to subsequent primary carers (such as adoptive parents). This has been observed in institutionalized populations (Chisholm et al. 1995; Chisholm 1998; Bruce et al. 2009; O’Connor et al. 2003) and foster children with RAD including DAD (Minnis et al. 2009). Anecdotal reports and observations of the current adoptive sample indicate that the majority of children had formed very clear and discriminating attachments to their adoptive parents. A second prediction could be that children adopted after this critical window would tend to have a rather different pattern of selective attachment difficulty – more in keeping with the disorganized patterns of attachment seen in children who remain in similar risk environments. This remains to be tested empirically.

Secondly, the apparently developmentally specific findings on the antecedents of DAD are in substantive contrast to the findings of the ERA study of a linear dose-response effect with time spent in institutional care with DAD (O’Connor et al. 1999; O’Connor et al. 2000; Rutter et al. 2007). It is worth noting that Kreppner et al. (2007) found a threshold effect of duration of institutionalization in relation to impairment across multiple domains of functioning including cognitive impairment, Autistic Spectrum Disorder and DAD, where multiple impairment was associated with deprivation lasting longer than 6 months. However, it is unclear how the cut-offs used to index impairment in this study relate to those used in the earlier studies reporting prevalence of DAD and the dose-response effect of deprivation. Clearly, the context in the ERA study is rather different to ours - the studied institutions were extremely depriving, with multiple caregivers, a lack of one-to-one care plus physical risk and malnourishment. It is possible that children who spent longer in the institution never had the opportunity to form a selective attachment relationship at all. In our current non-institutionalized sample, the lack of direct association between DAD and extent of adversity or other risk exposures, suggests a more complex developmental pathway to DAD, possibly also linked with genetic vulnerability.

Thirdly, an alternative conceptualization of DAD may be to view it as failure of emergence and persistent lack of stranger wariness, rather than disinhibition of care-seeking behavior. It has been argued that stranger wariness represents a developmental process, or behavioral system that interacts with, but is separable from attachment (Sroufe 1977). Longitudinal data suggests that stranger wariness also develops between 6 and 12 months, with limited increase between 12 and 24 months (Brooker et al. 2013; Emde et al. 1976). A potential hypothesis here, therefore, is that disruption in the formation of discriminated attachment relationships also disrupts the development of stranger wariness. This explanation would also be consistent with the persistence of disinhibited behavior in the context of secure and organized attachment patterns (Kreppner et al. 2011) and the dose-response effect of institutionalization observed in the ERA study. A recent fMRI study found that post-institutionalized children showed equivalent amygdala response to images of mothers and strangers, where non-institutionalized comparison children showed reduced activation to strangers. Lack of discrimination in response to strangers was associated with parent reports of indiscriminately friendly behavior as well as later age-at-adoption (Olsavsky et al. 2013). Further research in this area could involve longitudinal studies of the emergence of attachment and stranger wariness in adopted children and employ experimental and neurophysiological paradigms to investigate the underlying cognitive mechanisms of DAD.

A number of limitations to the study and the inferences possible from it need consideration. As mentioned above, recruitment was through families’ self-referral. Although the recruitment procedure avoided identification of prior hypotheses and the sample is comparable at least in key demographic factors, to nationally representative adoption cohorts (Selwyn et al. 2014), a degree of selection bias cannot be ruled out. The sample sizes are also modest and replication in large representative samples is clearly required. The cut-off used in triangulation of measures to determine prevalence of DAD was based on the distribution of continuous scores and results of reliability analysis. It does not equate to a clinical diagnosis of DAD or DSED; there are currently no gold-standard clinical diagnostic interviews for DSED in older children. Future work should aim to validate clinical thresholds on measures of DSED for use in research. Data on care and maltreatment history was based on detailed interview with adoptive parents and documentation they held: while details of timings into care will be accurate, data on adversity exposure prior to adoption is dependent on the extent of their knowledge. This may have impacted on ratings of severity of maltreatment. Confidence ratings were included in analysis using maltreatment severity to control for this. Nevertheless, future research should aim to incorporate direct examination of social services case files in order to capture more detailed information on the nature, timing and chronicity of maltreatment for more robust analysis of the aetiological mechanisms of DAD. The ED sample was selected to be low-risk with no experience of maltreatment or pre-natal adversity. It was not possible to gather independent data on maltreatment or pre-natal adversity within the ED group, but children with a history of local authority care, adoption and/or involvement with social services were excluded from the study. Clinicians screened all participants using these criteria. It is not possible to rule out that some of the ED sample may have experienced unreported maltreatment but our exclusion criteria were designed to minimize this possibility. Data on psychopathology was returned for only half of the ED sample despite attempts to prompt parents to complete the DAWBA. This was due to particular difficulty in maintaining engagement with this group following the initial home visit. There were however no significant differences in demographic factors or prevalence of DAD between ED participants with and without psychopathology data. None of the multi-informant measures of DAD could be made blind to group, but the triangulation methodology was designed to minimize an overall effect of response biases. We did not measure IQ but did have a standard measure of language; neither this nor parent reported learning difficulties were correlated with DAD outcome.

Our findings have significant clinical and practical implications. Adoption is the preferred approach in the UK to achieving permanence for children who cannot remain with birth families, but there is insufficient knowledge about subsequent developmental progress or outcomes, although placement breakdown and continued difficulties have been shown in a high proportion (in 23 % and 28 %, respectively) of late placed adoptees (Rushton and Dance 2006). More recent large-scale work on disruption of adoptive placements in England has shown that, whilst between three and eight percent of placements disrupt, there are high levels of mental health difficulty in adopted children; 25 % of children for whom parents report that the placement is going generally well and 97 % of those for whom the placement has disrupted scored above clinical thresholds on the Strengths and Difficulties Questionnaire (Selwyn et al. 2014). Our data complements this, in highlighting a significant amount of ongoing social impairment and morbidity in middle childhood despite a relatively limited length of exposure to post-natal adversity and subsequent years spent in a stable adoptive placement. In addition to DAD, 65 % of adopted children showed clinically significant psychopathology with substantial co-morbidity, comparable with rates of morbidity found in UK foster and residential care (Ford et al. 2007). There are real implications here for provision of post-adoptive support, particularly in the context of the UK governments drive to increase the number of children adopted from care. It will be important for future work to examine the possibility that DAD may predict functional adaptation, placement breakdown and eventual outcome as well as parental wellbeing in adoptive families and other forms of out-of-home placement. If the nature and prevalence of DAD found here, and its apparent relation to timing of initial placement is replicated on wider samples then there will be important implications for prevention and intervention within adoption practice.

## Electronic Supplementary Material

ESM 1 (DOCX 13 kb)

ESM 2. (DOCX 13 kb)

## References

[CR1] American Psychiatric Association (1980). Diagnostic and statistical manual of mental disorders.

[CR2] American Psychiatric Association (2013). Diagnostic and statistical manual of mental disorders.

[CR3] Barnett D, Manly JT, Cicchetti D, Cicchetti D, Toth S (1993). Defining child maltreatment: the interface between policy and research. *Advances in applied developmental psychology*: *child abuse*, *child development and social policy*.

[CR4] Botting N, Conti-Ramsden G (2008). The role of language, social cognition, and social skill in the functional social outcomes of young adolescents with and without a history of SLI. British Journal of Developmental Psychology.

[CR5] Brooker RJ, Buss KA, Lemery K, Aksan N, Davidson RJ, Goldsmith HH (2013). The development of stranger fear in infancy and toddlerhood: normative development, individual differences, antecedents, and outcomes. Developmental Science.

[CR6] Bruce J, Tarullo AR, Gunnar MR (2009). Disinhibited social behavior among internationally adopted children. Development and Psychopathology.

[CR7] Chisholm K (1998). A three year follow-up of attachment and indiscriminate friendliness in children adopted from Romanian orphanages. Child Development.

[CR8] Chisholm K, Carter MC, Ames EW, Morison SJ (1995). Attachment security and indiscriminately friendly behavior in children adopted from Romanian orphanages. Development and Psychopathology.

[CR9] Department for Education (2013). Children looked after in England (including adoption and care leavers) year ending 31 March 2013.

[CR10] Emde R, Gaensbauer T, Harmon R (1976). *Emotional expression in infancy*: *a biobehavioral study*. *Psychological issues monograph 37*.

[CR11] Ford T, Vostanis P, Meltzer H, Goodman R (2007). Psychiatric disorder among British children looked after by local authorities: comparison with children living in private households. The British Journal of Psychiatry: the Journal of Mental Science.

[CR12] Goodman R, Ford T, Simmons H, Gatward R, Meltzer H (2000). Using the strengths and difficulties questionnaire (SDQ) to screen for child psychiatric disorders in a community sample. British Journal of Psychiatry.

[CR13] Goodman R, Ford T, Richards H, Gatward R, Meltzer H (2000). The development and well-being assessment: description and initial validation of an integrated assessment of child and adolescent psychopathology. Journal of Child Psychology and Psychiatry.

[CR14] Kay C, Green J (2013). Reactive attachment disorder following early maltreatment: systematic evidence beyond the institution. Journal of Abnormal Child Psychology.

[CR15] Kay C, Green J (2015). Social cognitive deficits and biases in maltreated adolescents in UK out-of-home care: relation to disinhibited attachment disorder and psychopathology. Development and Psychopathology. Advance Online publication..

[CR16] Kreppner J, Kumsta R, Rutter M, Beckett C, Castle J, Stevens S (2010). IV. Developmental course of deprivation-specific psychological patterns: early manifestations, persistence to age 15, and clinical features. Monographs of the Society for Research in Child Development.

[CR17] Kreppner J, Rutter M, Beckett C, Castle J, Colvert E, Groothues C (2007). Normality and impairment following profound early institutional deprivation: a longitudinal follow-up into early adolescence. Developmental Psychology.

[CR18] Kreppner J, Rutter M, Marvin R, O’Connor T, Sonuga-Barke E (2011). Assessing the concept of the ‘insecure-other’ category in the Cassidy Marvin scheme: changes between 4 and 6 years in the English and Romanian adoptee study. Social Development.

[CR19] Kumsta R, Kreppner J, Rutter M, Beckett C, Castle J, Stevens S, Sonuga-Barke E (2010). III. Deprivation-specific psychological patterns. Monographs of the Society for Research in Child Development.

[CR20] Lyons-Ruth K, Bureau JF, Riley CD, Atlas-Corbett AF (2009). Socially indiscriminate attachment behavior in the strange situation: convergent and discriminant validity in relation to caregiving risk, later behavior problems, and attachment insecurity. Development and Psychopathology.

[CR21] Meltzer H, Gatward R, Goodman R, Ford T (2000). M*ental health of children and adolescents in Great Britain*.

[CR22] Minnis H, Reekie J, O’Connor T, Roland A, Gray A, Plomin R (2007). Genetic, environmental and gender influences on attachment disorder behaviors. British Journal of Psychiatry.

[CR23] Minnis H, Green J, O’Connor TG, Liew A, Glaser D, Taylor E (2009). An exploratory study of the association between reactive attachment disorder and attachment narratives in early school-age children. Journal of Child Psychology and Psychiatry.

[CR24] O’Connor TG, Bredenkamp D, Rutter M (1999). Attachment disturbances and disorders in children exposed to early severe deprivation. Infant Mental Health Journal.

[CR25] O’Connor TG, Rutter M, the English and Romanian Adoptees Study Team (2000). Attachment disorder behavior following early severe deprivation: Extension and longitudinal follow-up. Journal of the American Academy of Child and Adolescent Psychiatry.

[CR26] O’Connor TG, Marvin RS, Rutter M, Olrick JT, Britner PA (2003). Child-parent attachment following early institutional deprivation. Development and Psychopathology.

[CR27] Olsavsky AK, Telzer EH, Shapiro M, Humphreys KL, Flannery J, Godd B (2013). Indiscriminate amygdala response to mothers and strangers after early maternal deprivation. Biological Psychiatry.

[CR28] Pears KC, Bruce J, Fisher PA, Kim HK (2010). Indiscriminate friendliness in maltreated foster children. Child Maltreatment.

[CR29] Pritchett R, Pritchett J, Marshall E, Davidson C, Minnis H (2013). Reactive attachment disorder in the general population: a hidden ESSENCE disorder. The Scientific World Journal..

[CR30] Rushton A, Dance C (2006). The adoption of children from public care: a prospective study of outcome in adolescence. Journal of the American Academy of Child and Adolescent Psychiatry.

[CR31] Rutter M, Colvert E, Kreppner J, Beckett C, Castle J, Groothues C (2007). Early adolescent outcomes for institutionally deprived and non-deprived adoptees. I: disinhibited attachment. Journal of Child Psychology and Psychiatry.

[CR32] Scott S, Sylva K, Beckett C, Kallitsoglou A, Doolan M, Ford T (2012). Should parenting programmes to improve children’s life chances address child behavior, reading skills, or both? Rationale for the helping children achieve trial. The European Journal of Developmental Psychology.

[CR33] Selwyn J, Wijedasa D, Meakings S (2014). *Beyond the adoption order*: *challenges*, *interventions and adoption disruption*.

[CR34] Semel E, Wiig E, Secord W (2003). *Clinical evaluation of language fundamentals*, fourth edition (CELF-4).

[CR35] Sroufe A (1977). Wariness of strangers and the study of infant development. Child Development.

[CR36] Tizard B, Rees J (1975). The effect of early institutional rearing on the behavior problems and affectional relationships of four-year-old children. Journal of Child Psychology and Psychiatry.

[CR37] World Health Organization (1992). *The ICD-10 classification of mental and behavioral disorders*: *clinical descriptions and diagnostic guidelines*.

[CR38] Zeanah, C.H., & Gleason, M.M. (2010). Reactive attachment disorder: a review for DSM-V. American Psychiatric Association. Retrieved from http://www.researchgate.net/profile/Charles_Zeanah/publication/228683818_Reactive_attachment_disorder_A_review_for_DSM-V/links/0deec51e86576d1e8c000000.pdf.

