# Supplementary material for: Disinhibited Attachment Disorder in UK Adopted Children During Middle Childhood: Prevalence, Validity and Possible Developmental Origin
Source: J Abnorm Child Psychol. 2016 Feb 9;44(7):1375–86. doi: 10.1007/s10802-016-0131-2 (PMC5007266; doi:10.1007/s10802-016-0131-2)
Supplement: Supplementary file 1 — (DOCX 13 kb) [file 10802_2016_131_MOESM1_ESM.docx]

Supplementary Table 1: Correlation between CAPA-RAD, RPQ and Observation measures of DAD.

| Measure |  | CAPA-RAD | RPQ | Observation |
| --- | --- | --- | --- | --- |
| CAPA-RAD | Pearson Correlation (*p*) | ~ | .464 (.000) | .539 (.000) |
| RPQ | Pearson Corrleation (*p*) | ~ | ~ | .279 (.000) |
