# Supplementary material for: Disinhibited Attachment Disorder in UK Adopted Children During Middle Childhood: Prevalence, Validity and Possible Developmental Origin
Source: J Abnorm Child Psychol. 2016 Feb 9;44(7):1375–86. doi: 10.1007/s10802-016-0131-2 (PMC5007266; doi:10.1007/s10802-016-0131-2)
Supplement: Supplementary file 2 — (DOCX 13 kb) [file 10802_2016_131_MOESM2_ESM.docx]

Supplementary Table 2: T-tests showing significant differences in mean CAPA-RAD, RPQ and Observation scores according to presence of triangulated DAD.

| Measure | DAD | No DAD | *t* | *df* | Sig. |
| --- | --- | --- | --- | --- | --- |
|  | Mean (SD) | Mean (SD) |  |  |  |
| CAPA-RAD Total | 3.81 (1.86) | .873 (1.40) | -8.30 | 45.2 | .000 |
| RPQ Total | 2.27 (1.94) | .407 (.958) | -4.96 | 33.0 | .000 |
| Observation Total | 2.96 (2.02) | .290 (.745) | -7.20 | 32.7 | .000 |
